# Integrative single-cell and bulk transcriptomic analyses identify a microglia-associated NAGLU signature linked to disulfidptosis-associated transcriptional patterns after spinal cord injury

**DOI:** 10.3389/fimmu.2026.1899070

**Published:** 2026-08-14

**Authors:** Xiaoqin Liu, Jiating Hu, Wenxia Zhu, Guodong Shi, Ping Kang

**Affiliations:** 1 Yan’an Medical College of Yan’an University, Yan’an, China; 2 Department of Neurosurgery, The Affiliated Hospital of Yan’an University, Yan’an, China

**Keywords:** disulfidptosis, microglia, NAGLU, single-cell RNA sequencing, spinal cord injury

## Abstract

**Background:**

Spinal cord injury (SCI) induces profound neuroinflammatory responses and extensive cellular remodeling within the injured spinal cord. Disulfidptosis, a recently identified form of regulated cell death associated with disulfide stress, has been implicated in cellular stress responses and tissue injury. However, the transcriptional characteristics of disulfidptosis-related genes (DRGs) and their associations with the cellular microenvironment of SCI remain poorly understood.

**Methods:**

Single-cell RNA sequencing data from GSE162610 were analyzed to characterize cellular heterogeneity in DRG-associated transcriptional signatures. AUCell was used to calculate DRG signature scores at the single-cell level, and differentially expressed genes associated with DRG signatures were identified by comparing cells with high and low DRG signature scores. Bulk transcriptomic data from GSE47681 were analyzed using differential expression analysis and weighted gene co-expression network analysis (WGCNA) to identify SCI-associated differentially expressed genes and gene modules correlated with DRG signature scores. Candidate genes were identified by integrating single-cell marker genes, bulk differentially expressed genes, and genes from DRG signature-associated WGCNA modules. A random forest algorithm was applied to prioritize key candidate genes, followed by validation in the independent dataset GSE45006. The expression pattern of the selected candidate gene was further examined at single-cell resolution and validated at the protein level in a rat SCI model 3 days after injury.

**Results:**

DRG signature scores exhibited marked heterogeneity across spinal cord cell types. Integrative analyses identified 24 candidate genes associated with DRG-related transcriptional alterations. Functional enrichment analyses highlighted lysosome-related pathways, efferocytosis, macrophage activation, and glycosaminoglycan degradation. Random forest analysis prioritized three genes for external validation, among which *Naglu* showed consistent differential expression in the independent dataset. Single-cell analysis indicated that *Naglu* was predominantly expressed in microglia. Protein-level validation further demonstrated increased NAGLU expression in spinal cord tissues from SCI rats at 3 days post-injury compared with Sham controls.

**Conclusions:**

This study characterizes DRG-associated transcriptional heterogeneity in SCI and identifies *Naglu* as a microglia-associated lysosomal gene upregulated after spinal cord injury. These findings suggest that DRG-associated transcriptional states are linked to microglial lysosomal responses after SCI and provide a basis for future mechanistic studies investigating neuroinflammation and tissue repair.

## 1 Introduction

Following spinal cord injury (SCI), patients commonly develop severe neurological deficits affecting movement, sensation, and autonomic control beneath the injured segment, resulting in substantial reductions in quality of life and imposing significant socioeconomic and psychological burdens (1, 2). SCI pathology is generally divided into a primary injury phase and a subsequent secondary damage phase (3). The primary injury is caused by acute physical trauma, commonly arising from traffic collisions, accidental falls, or athletic injuries (4). In contrast, secondary injury involves a series of progressive pathological events, including edema formation, hemorrhagic damage, neuroinflammation, redox imbalance, impaired mitochondrial activity, excitotoxicity, and diverse regulated cell death pathways (5). Although substantial progress has been made, the molecular mechanisms underlying these regulated cell death pathways during secondary SCI remain incompletely understood, limiting the identification of effective therapeutic targets (6).

Disulfidptosis is a recently identified form of regulated cell death associated with excessive intracellular disulfide accumulation and cytoskeletal disruption under metabolic stress (7). Recent studies indicate that disulfidptosis is associated with disturbances in redox homeostasis and cellular metabolism, particularly under conditions of elevated SLC7A11 expression and reduced NADPH availability (8–11). Given the prominent oxidative stress and metabolic disturbances observed after SCI, disulfidptosis-associated molecular signatures may provide insights into stress-related cellular responses during SCI progression. However, the transcriptional characteristics of disulfidptosis-related genes (DRGs) and their cell-type-specific distribution in SCI remain largely unexplored.

Here, we systematically integrated publicly available single-cell and bulk transcriptomic datasets to investigate cell type-specific transcriptional patterns associated with DRG signatures SCI. By combining single-cell transcriptomic profiling, bulk differential expression analysis, and weighted gene co-expression network analysis (WGCNA), we identified a candidate gene set associated with DRG signature-related transcriptional alterations. Candidate genes were subsequently prioritized using a random forest (RF)-based feature ranking approach and externally validated in the independent GSE45006 dataset. This integrative analytical framework provides a comprehensive characterization of DRG signature-associated transcriptional alterations in SCI and identifies candidate genes for future mechanistic and functional investigations.

## 2 Methods

### 2.1 Data collection

We retrieved the gene expression datasets GSE162610, GSE47681, and GSE45006 from the Gene Expression Omnibus (GEO) database (https://www.ncbi.nlm.nih.gov/geo/) (12). For the bulk transcriptomic analyses, only samples collected at 1, 3, and 7 days post-injury (dpi) were included to maintain consistency across datasets. To identify genes consistently associated with the acute phase of SCI, samples from these three post-injury time points were pooled into a single SCI group for downstream analyses rather than analyzed separately.

GSE162610 contains single-cell RNA sequencing (scRNA-seq) profiles generated from 10 spinal cord samples, including three uninjured controls and seven SCI samples collected at 1, 3, and 7 days post-injury (13). To comprehensively characterize the cellular landscape following SCI, all available single-cell samples were integrated and analyzed as a pooled dataset without stratification by experimental group or post-injury time point.

GSE47681 (GPL1261) contains microarray expression profiles derived from adult male C57BL/6J mice. After excluding trkB.T1 knockout samples, 16 wild-type samples were included, comprising four Sham samples and twelve SCI samples, with four biological replicates collected at each of 1, 3, and 7 days post-injury (14). The SCI samples from all three time points were pooled and analyzed as a single acute SCI group.

GSE45006 (GPL1355) contains microarray expression profiles generated using the Affymetrix Rat Genome 230 2.0 platform. The complete dataset comprises 24 samples, including four Sham samples and 20 SCI samples collected at 1 day, 3 days, 1 week, 2 weeks, and 8 weeks post-injury, with four biological replicates at each post-injury time point. To maintain consistency with the discovery dataset, only the 12 SCI samples collected at 1, 3, and 7 days post-injury and the four Sham samples were included for external validation. The SCI samples from the three selected time points were pooled into a single SCI group and compared with the Sham group. The sex of the animals was not reported in the original study.

### 2.2 scRNA-seq data preprocessing and downstream analysis

Raw expression count matrices were processed using the “Seurat” package in R (version 5.1.0) (15). Quality control procedures were applied to remove low-quality genes and cells. Genes detected in fewer than 10 cells were removed. Cells with fewer than 200 detected genes, mitochondrial transcript proportions >20%, log10GenesPerUMI ≤ 0.8, or total UMI counts <500 or >6,000 were excluded. Doublets were identified and filtered using the scDblFinder method implemented in the “SingleCellExperiment” package. Cell cycle scores were calculated based on canonical S-phase and G2/M-phase gene signatures. Data normalization and variance stabilization were performed using SCTransform, with mitochondrial content and cell cycle-related variables (S.Score, G2M.Score, CC.Difference) regressed out. To minimize technical heterogeneity among samples, batch correction was performed using ‘Harmony’. PCA was performed on the Harmony-integrated expression matrix, followed by clustering analysis using Seurat functions FindNeighbors and FindClusters. After evaluating multiple clustering resolutions, a resolution of 0.8 was selected for downstream analyses. t-SNE was used for visualization of cellular distributions. Cluster-specific marker genes were identified using Seurat’s FindAllMarkers function (min.pct = 0.1, logfc.threshold = 0.5). Genes with adjusted *P* values <0.05 and average log2 fold changes >0.5 were considered significant. DRG signature scores were subsequently calculated at the single-cell level using AUCell based on the predefined DRG gene set.

### 2.3 Identification of disulfidptosis-associated genes at the single-cell level

A total of 39 previously reported disulfidptosis-related genes (DRGs) were collected from published studies (**Table 1**) (7, 16–18). Because the transcriptomic datasets analyzed in this study were generated from rodents, the reported human DRGs were converted to their corresponding rodent orthologs before downstream analyses. Gene symbols were subsequently presented according to the official rodent gene nomenclature throughout the bioinformatic analyses, whereas protein names were presented in uppercase according to standard protein nomenclature. Cluster-specific marker genes overlapping with the predefined DRG list were identified and retained for subsequent analyses. Using the predefined 39 DRGs, AUCell was applied to calculate DRG signature scores at the single-cell level. AUCell evaluates the relative enrichment of a predefined gene set within individual cells based on gene expression ranking and does not directly infer biological activity or account for the directionality or functional contribution of individual genes. Cells were subsequently stratified into high- and low-score groups according to the median AUCell score, which was used as an exploratory threshold for relative comparison of DRG signature enrichment rather than to define biologically distinct cellular states. To evaluate whether the DRG signature score was influenced by general stress-related transcriptional programs, a heat shock protein-related gene (HSP-related) signature was additionally constructed based on previously reported heat shock protein-associated genes (19). The same AUCell algorithm was applied to calculate the heat shock protein-related gene signature score at the single-cell level for comparison with the DRG signature score.

**Table 1:** Disulfidptosis-related genes used in this study.

Gene set	Genes
Disulfidptosis-related genes	ABI2; ACSL4; ACTB; ACTN4; BAK1; CAPZB; CD2AP; CYFIP1; DSTN; FLNA; FLNB; FLNC; GYS1; INF2; IQGAP1; LRPPRC; MYH10; MYH9; MYL6; NADPH; NCKAP1; NDUFA11; NDUFS1; NUBPL; OXSM; PDLIM1; PRDX1; RAC1; RBK1; RPN1; SLC3A2; SLC7A11; TLN1; TLN2; WASF2; MYL6B; DBN1; ACTN1; MYH11

### 2.4 Differential expression analysis

Differential expression analysis was performed using the GSE47681 dataset. Differentially expressed genes (DEGs) between SCI and Sham groups were identified using the “limma” package in R. Expression data were normalized prior to differential expression analysis. Multiple testing correction was performed using the Benjamini–Hochberg method to control the false discovery rate (FDR). Genes with an adjusted *P*-value < 0.05 and |log_2_ fold change| > 1 were considered differentially expressed. Volcano plots were generated using the “ggplot2” package to visualize differential expression patterns.

### 2.5 Disulfidptosis-related gene signature scoring

To characterize the overall transcriptional patterns of the predefined 39 DRGs across bulk transcriptomic samples, principal component analysis (PCA) was performed using the expression profiles of the predefined DRG set. The first principal component was extracted to construct a composite DRG signature score for each sample, representing the overall transcriptional variation of the DRG set. The resulting score was used as a transcriptomic signature for subsequent association analyses and should be interpreted as a gene signature score rather than direct evidence of disulfidptosis.

### 2.6 WGCNA

Gene co-expression networks were constructed using the GSE47681 bulk transcriptome dataset. Genes with low variability were excluded, and the top 25% most variable genes were selected for the primary network construction. Pearson correlation coefficients were calculated to generate a similarity matrix, which was transformed into a weighted adjacency matrix using a soft-thresholding power (β = 13) selected based on a balance between scale-free topology fitting and network connectivity. The topological overlap matrix (TOM) was subsequently calculated, followed by hierarchical clustering and dynamic tree cutting to identify co-expression modules. A minimum module size of 100 genes was applied, and module eigengenes (MEs) were calculated as the first principal component of each module. The primary objective of WGCNA was to identify gene co-expression modules associated with the DRG signature score rather than to define functional or mechanistic disulfidptosis modules. Modules showing significant Pearson correlations between their eigengenes and the DRG signature score (*P* < 0.05) were defined as DRG signature-associated co-expression modules and were selected for downstream functional enrichment analysis and candidate gene identification.

To assess the robustness of the gene filtering strategy, sensitivity analyses were performed by reconstructing independent WGCNA networks using the top 15%, 25%, and 50% most variable genes. The robustness of module identification across different filtering strategies was evaluated using gene-level overlap, Jaccard index, and absolute module–trait correlations.

### 2.7 RF-based prioritization of candidate genes identified by integrative transcriptomic analysis

Statistical analysis and visualization were performed in R using the packages “randomForest”, “caret”, and “ggplot2”. To further prioritize candidate genes identified through integrative transcriptomic analyses in SCI, a RF model was applied to the expression profiles of a biologically pre-filtered candidate gene set derived from the intersection of (1) genes differentially expressed between SCI and Sham samples, (2) genes differentially expressed between high- and low-DRG signature cells, and (3) genes belonging to DRG signature-associated WGCNA modules. Importantly, the RF model was not used for *de novo* gene discovery or as an independent diagnostic classifier, but rather as a feature prioritization approach within a predefined candidate gene space. Model performance was internally evaluated using repeated 5-fold cross-validation (5 folds × 50 repeats). Performance metrics, including ROC-AUC, sensitivity, specificity, and accuracy, were summarized in **Supplementary Table 2**. Gene importance was assessed using the Mean Decrease Gini index, representing the relative importance of each candidate gene in SCI versus Sham classification. Candidate genes were ranked according to their importance scores, and the top 10 genes were visualized using bar plots. Among the top-ranked genes, the candidate showing the most consistent evidence from RF prioritization and independent transcriptomic validation was selected for further experimental investigation. The RF-derived ranking was interpreted as a measure of relative feature importance rather than direct evidence of causal involvement in disulfidptosis. Detailed model configuration and internal validation performance metrics are provided in **Supplementary Tables 1**, **2**.

### 2.8 Functional enrichment analysis

Gene Ontology (GO) and Kyoto Encyclopedia of Genes and Genomes (KEGG) pathway enrichment analyses were performed using the “ClusterProfiler” package to characterize the biological functions and signaling pathways associated with the identified candidate genes (20). Gene symbols were converted into Entrez Gene IDs using the bitr function. GO enrichment analysis was conducted across three functional categories, including biological process (BP), cellular component (CC), and molecular function (MF). Statistical significance was determined based on an adjusted *P* value < 0.05 after Benjamini–Hochberg correction for multiple testing. KEGG pathway enrichment analysis was performed using the same criteria. The enrichment results were visualized using bubble plots, in which bubble size represents the number of enriched genes and color intensity indicates the adjusted *P* values.

### 2.9 Establishment of SCI model rats

Adult female Sprague–Dawley rats (220–250 g) were purchased from the Laboratory Animal Center of Xi’an Jiaotong University (license No. SCXC (Shan) 2023–002). All animal procedures were approval by the Animal Ethics Committee of Yan’an University and performed in accordance with institutional guidelines. Animals were housed under standard laboratory conditions and randomly assigned to either the Sham or SCI group (n = 6 per group). Following intraperitoneal anesthesia with 2% sodium pentobarbital (30 mg/kg), rats were placed in the prone position under sterile conditions. A midline dorsal incision was made to expose the T9–T11 vertebrae, followed by laminectomy at the T10 level to expose the dura mater. In the SCI group, a calibrated impact (200 kdy) was delivered to the T10 spinal cord using a HI-0400 spinal cord impactor (PSI) to establish the SCI model. Successful injury was confirmed by immediate tail flick, bilateral hindlimb spasm, and local hemorrhage at the impact site with an intact dura mater. Rats in the Sham group underwent the identical surgical procedure without spinal cord impact. Postoperative care included intramuscular administration of penicillin sodium (8 × 10^4^ U/day) for three consecutive days and manual bladder expression twice daily until spontaneous voiding recovered. Spinal cord tissues centered on the lesion site were collected exclusively at 3 days post-injury for subsequent molecular and histological analyses. This time point was selected for experimental validation, whereas the bioinformatic analyses were performed using pooled public transcriptomic datasets from 1, 3, and 7 days post-injury.

### 2.10 Western Blot Analysis

Spinal cord tissues were lysed in RIPA lysis buffer supplemented with protease inhibitors. Protein concentrations were determined using the BCA Protein Assay system (Yamei, Shanghai, China). Equal amounts of protein (20 μg per sample) were separated by SDS–PAGE and transferred onto PVDF membranes. After blocking with 5% skim milk in TBST for 2 h at room temperature, membranes were incubated overnight at 4 °C with primary antibodies against NAGLU (1:1000, Abcam, ab214671) and GAPDH (1:5000, Proteintech, 60004-1-Ig) antibodies. After washing with TBST, membranes were incubated with HRP-conjugated goat anti-rabbit IgG secondary antibody (1:5000) for 1 h at room temperature. Protein bands were visualized using an enhanced chemiluminescence (ECL) detection system, and relative protein expression levels were quantified by densitometric analysis.

### 2.11 Immunofluorescence staining

After deep anesthesia, rats were perfused transcardially with PBS followed by 4% paraformaldehyde (PFA) fixation. Spinal cord segments containing the injury epicenter were dissected, post-fixed in 4% PFA overnight at 4 °C, and subsequently cryoprotected in 30% sucrose solution until complete cryoprotection was achieved. The tissues were embedded in OCT compound and cryosectioned into 10 μm-thick sections. For immunofluorescence staining, sections were fixed with 4% PFA, washed with PBS, permeabilized with 0.3% Triton X-100, and blocked with normal goat serum. The sections were incubated overnight at 4 °C with primary antibody against NAGLU (1:100, Abcam, ab214671), followed by incubation with an appropriate fluorescently labeled secondary antibody for 1 h at room temperature in the dark. Nuclei were counterstained with DAPI-containing anti-fade mounting medium (Solarbio, S2110). Fluorescence images were captured using a fluorescence microscope.

### 2.12 Statistical analysis

R software (v4.1.1) together with GraphPad Prism v8.0.0 were used for statistical processing. Animal experiments were performed using six rats per group across three independent experimental batches, with two animals included per group in each batch. Normality was assessed using the Shapiro–Wilk test, and homogeneity of variance was evaluated using the Brown–Forsythe test. Comparisons between two experimental groups were performed using two-tailed unpaired Student’s t-tests when parametric assumptions were satisfied. For datasets that violated parametric assumptions, the Mann–Whitney U test was used as a non-parametric alternative. Quantitative data are presented as mean ± SD, and statistical significance was defined as *P* < 0.05. In figures, one, two, and three asterisks indicate *P* < 0.05, *P* < 0.01, and *P* < 0.001, respectively.

## 3 Results

### 3.1 Single-cell landscape of SCI

**Figure 1:**
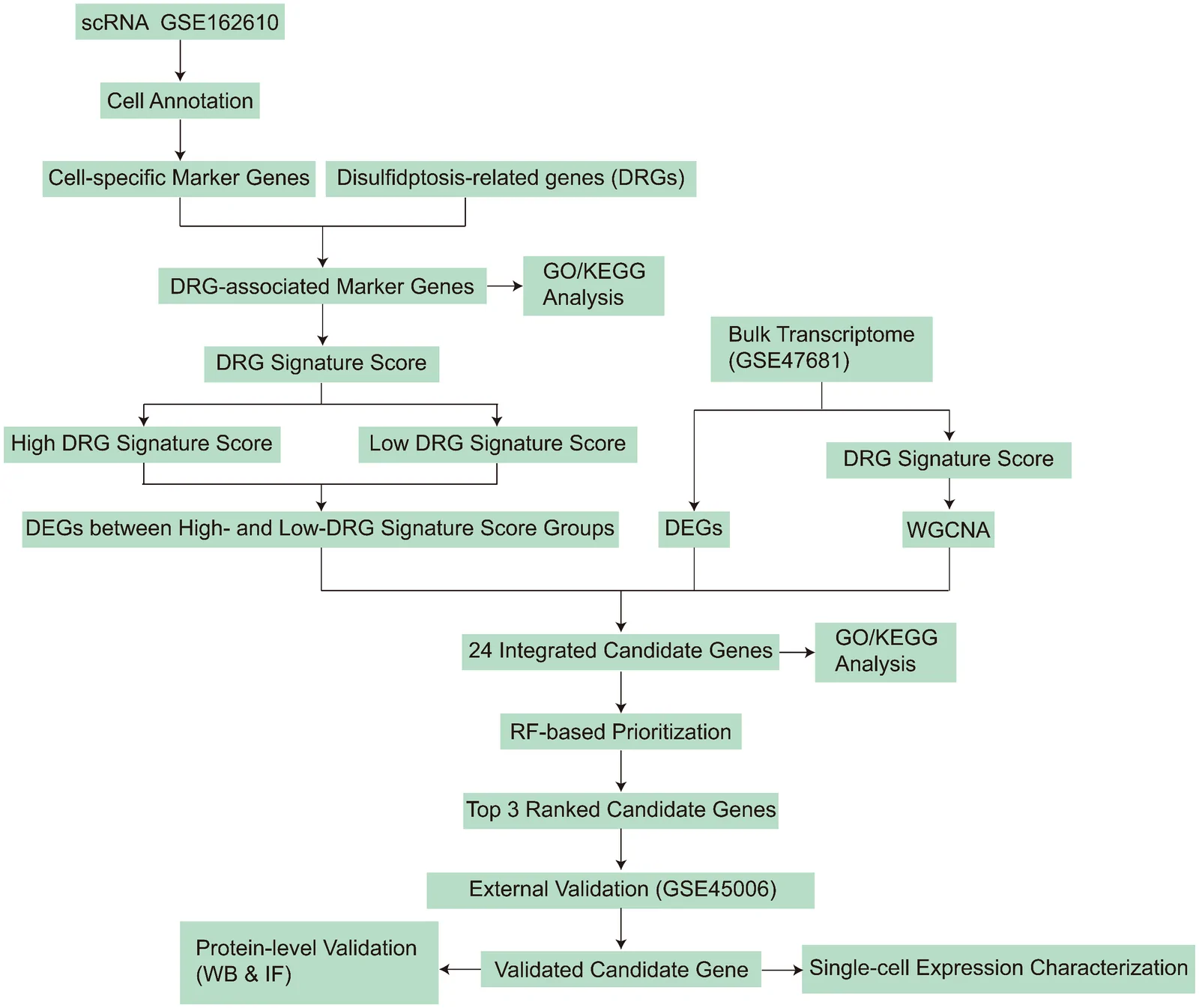
Overview of the experimental framework and bioinformatics analysis pipeline used in this study.

**Figure 2:**
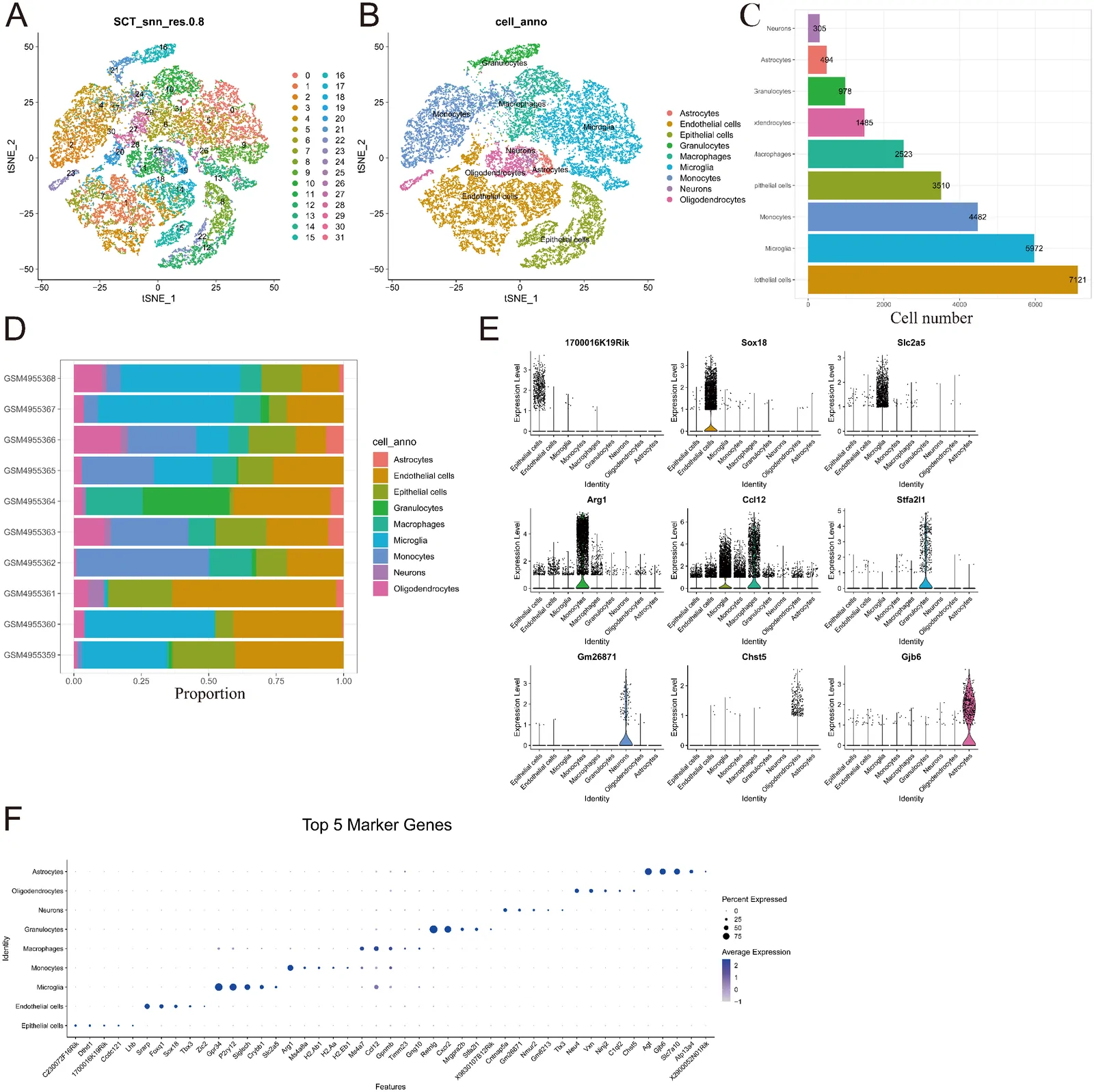
Identification and annotation of major cell types in the integrated scRNA-seq dataset. **(A)** t-SNE visualization showing 31 distinct cell clusters. **(B)** Annotation of major cell types based on SingleR predictions and canonical marker gene expression. **(C)** Number of cells in each annotated type. **(D)** Relative proportions of annotated cell types across individual samples. **(E)** Violin plots showing the expression of representative canonical marker genes across annotated cell types. **(F)** Dot plot showing the top five marker genes identified for each annotated cell type.

The overall study design and analytical workflow are illustrated in **Figure 1**. After quality control, a total of 29,791 cells from the GSE162610 dataset were retained for downstream analysis. To characterize the cellular landscape of spinal cord injury at single-cell resolution, all samples, including both uninjured and SCI tissues collected at multiple post-injury time points, were integrated and analyzed as a pooled dataset without stratification by experimental group or time point. Unsupervised clustering using t-SNE identified 31 distinct cell clusters (**Figure 2A**). Cell type annotation was performed by integrating SingleR-based predictions with canonical marker gene expression. Based on this combined strategy, the clusters were classified into nine major cell types, including astrocytes, endothelial cells, epithelial cells, granulocytes, macrophages, microglia, monocytes, neurons, and oligodendrocytes (**Figure 2B**). The distribution and relative proportions of major cell types across the integrated dataset are shown in **Figures 2C, D**, reflecting the overall cellular composition of the spinal cord injury microenvironment. To further characterize the temporal features of the single-cell dataset, the cellular composition was additionally evaluated across different post-injury time points (Uninjured, 1 dpi, 3 dpi, and 7 dpi). The proportions of major cell populations exhibited temporal changes across different post-injury stages, and t-SNE visualization demonstrated the temporal distribution of annotated cell populations during SCI progression (**Supplementary Figures 1A–C**). Representative canonical marker genes displayed cell type-specific expression patterns consistent with their corresponding annotations, providing additional support for the reliability of cell type identification (**Figure 2E**). Furthermore, the top five highly expressed marker genes for each annotated cell population are summarized in **Figure 2F**.

### 3.2 Identification of DRGs at the single-cell level

**Figure 3:**
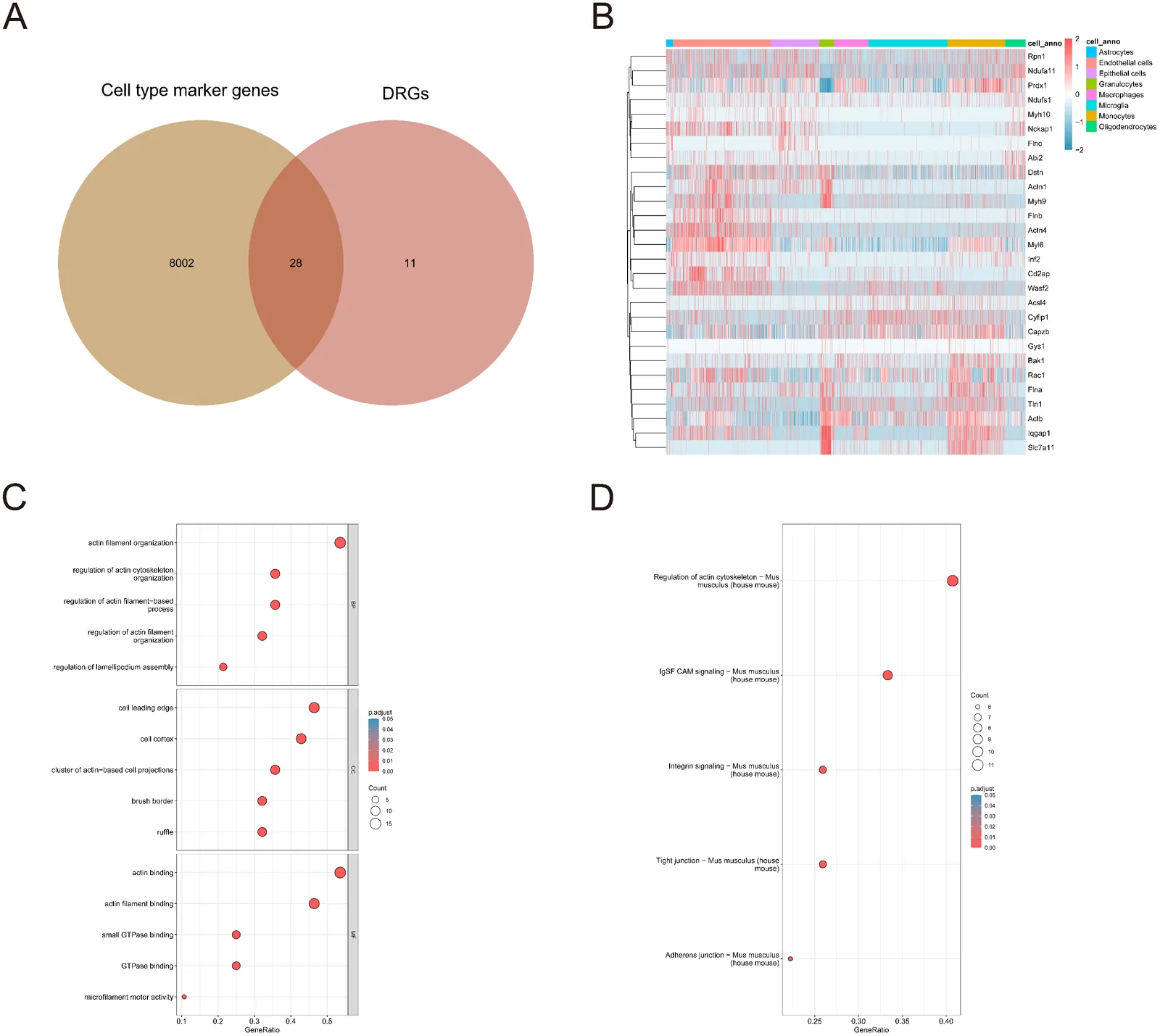
Single-cell expression patterns and functional enrichment analysis of DRGs. **(A)** Venn diagram showing the overlap between 39 DRGs and cell type-specific marker genes. **(B)** Heatmap showing the expression patterns of the 28 overlapping genes across annotated cell types. **(C)** GO enrichment analysis of the 28 overlapping genes. **(D)** KEGG pathway enrichment analysis of the 28 overlapping genes.

To identify the cell populations in which DRGs are preferentially expressed and to characterize their cell type-specific expression patterns within the SCI microenvironment, we performed an intersection analysis between DRGs and cell type-specific marker genes, identifying 28 overlapping genes (**Figure 3A**). Compared with bulk transcriptomic analysis, single-cell RNA sequencing enables the identification of the specific cellular context in which DRGs are expressed, thereby providing insights into their potential biological roles during SCI. The expression patterns of these genes across major cell populations are shown in **Figure 3B**, revealing distinct cell type–specific expression signatures. Notably, several genes, including *Iqgap1*, *Slc7a11*, *Actn1*, and *Myh9*, were predominantly enriched in granulocytes, whereas *Prdx1* exhibited relatively lower expression in this cell type, suggesting heterogeneous involvement of DRGs across immune-related populations. These findings indicate that DRGs exhibit marked cell type-specific expression patterns within the SCI microenvironment, highlighting the value of single-cell analysis in identifying the cellular context of disulfidptosis-related gene expression. In addition, the expression patterns of DRGs were further examined across different post-injury time points (Uninjured, 1 dpi, 3 dpi, and 7 dpi). As shown in **Supplementary Figure 1D**, the expression levels of these genes varied among different post-injury stages. To further explore the potential biological functions of these genes, GO enrichment analysis revealed significant enrichment in biological processes related to actin cytoskeleton organization (**Figure 3C**). KEGG pathway analysis further indicated that these genes were involved in cytoskeleton dynamics as well as cell–cell and cell–extracellular matrix adhesion pathways (**Figure 3D**). Given that cytoskeletal remodeling and immune cell adhesion are critical processes in inflammatory activation and infiltration following spinal cord injury, these findings suggest that DRGs may participate in regulating immune cell behavior and structural remodeling within the injured spinal cord microenvironment.

### 3.3 Scoring of disulfidptosis-related gene signature across cell types

**Figure 4:**
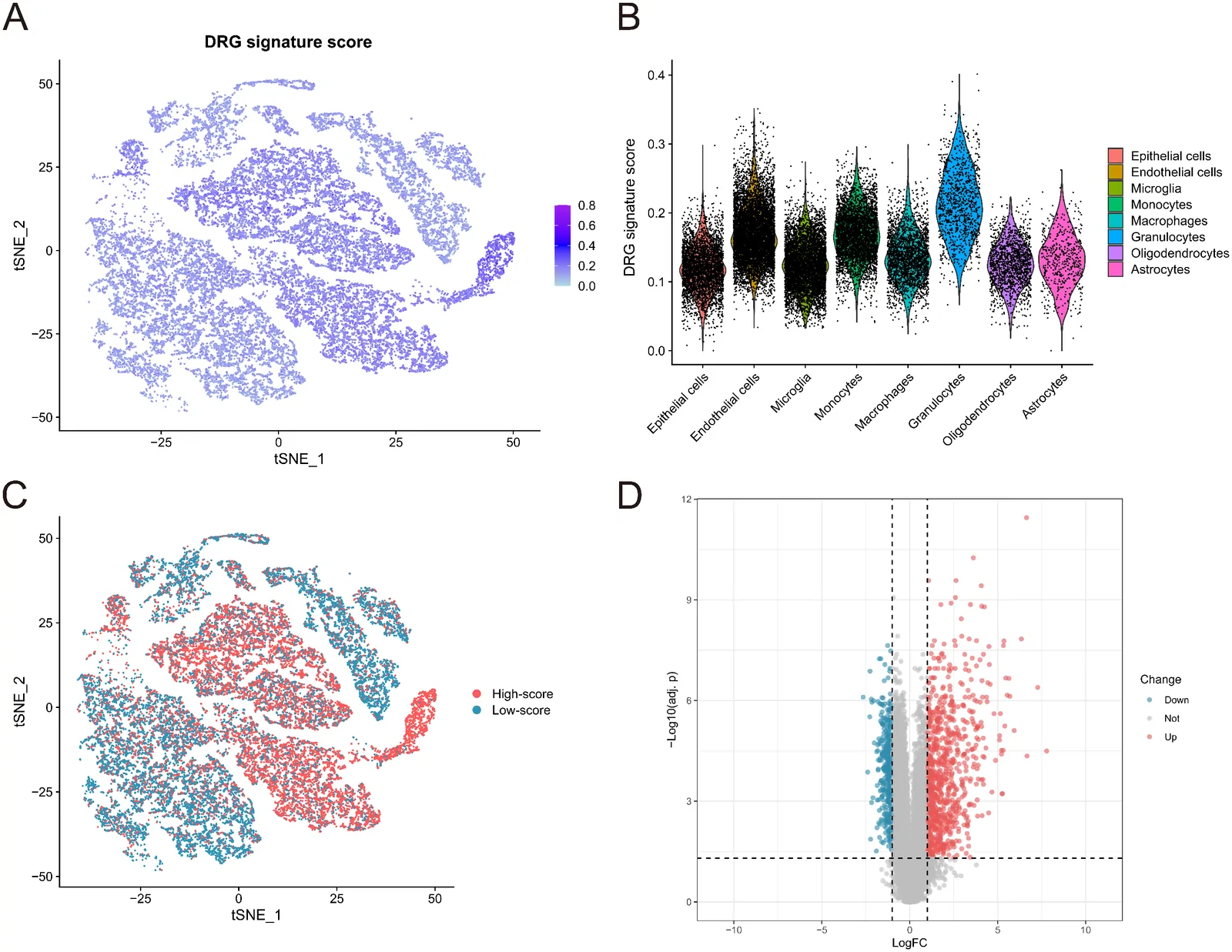
Distribution of the disulfidptosis-related gene signature score across cell types. **(A)** t-SNE visualization of disulfidptosis-related gene signature scores across single cells. **(B)** Violin plots showing the distribution of disulfidptosis-related gene signature scores across annotated cell types. **(C)** t-SNE visualization of cells stratified into high- and low-score groups based on the median gene signature score. **(D)** Volcano plot showing DEGs identified from all expressed genes between SCI and Sham samples.

Using the expression patterns of 39 DRGs, AUCell was applied to calculate a disulfidptosis-related gene signature score for each cell. AUCell estimates the relative enrichment of a predefined gene set among highly expressed genes within individual cells and does not directly reflect biological activity or confirm the occurrence of disulfidptosis. The t-SNE plot illustrates the distribution of gene signature scores across all cells (**Figure 4A**), showing heterogeneous enrichment patterns among different cell populations. Comparative analysis across annotated cell types revealed relatively higher gene signature scores in granulocytes and endothelial cells, whereas neurons exhibited lower scores (**Figure 4B**), suggesting cell type–specific variation in the expression of DRGs. Cells were stratified into high- and low-score groups based on the median gene signature score for exploratory visualization rather than defining biologically distinct cellular states, and the distribution of these groups is shown in **Figure 4C**. To provide complementary transcriptomic evidence at the tissue level, differential expression analysis was performed using the bulk transcriptomic dataset GSE47681. DEGs between SCI and Sham groups were identified using all expressed genes with thresholds of |log2FC| > 1.5 and adjusted *P* < 0.05. A total of 821 DEGs were identified, including 659 upregulated and 162 downregulated genes in SCI compared with Sham samples. The results are presented as a volcano plot (**Figure 4D**). This analysis was performed to characterize global transcriptional alterations associated with SCI and to provide an independent transcriptomic context for interpreting the single-cell-derived disulfidptosis-related gene signature. Therefore, the DEG analysis serves as complementary transcriptomic evidence for integrative interpretation rather than direct validation of disulfidptosis or the AUCell-derived gene signature score.

To further assess whether the DRG signature score was influenced by general stress-related transcriptional programs, we performed a comparative analysis using an HSP-related gene signature as an external stress-associated reference. Using the same AUCell-based approach, HSP-related signature scores were calculated across individual cells. A positive correlation was observed between DRG signature scores and HSP-related signature scores (Pearson r = 0.94, *P* < 2.2 × 10^−16^), indicating that DRG signatures share certain stress-associated transcriptional features with general stress responses (**Supplementary Figure 2**).

### 3.4 Identification of DRG signature–associated co-expression modules through WGCNA

**Figure 5:**
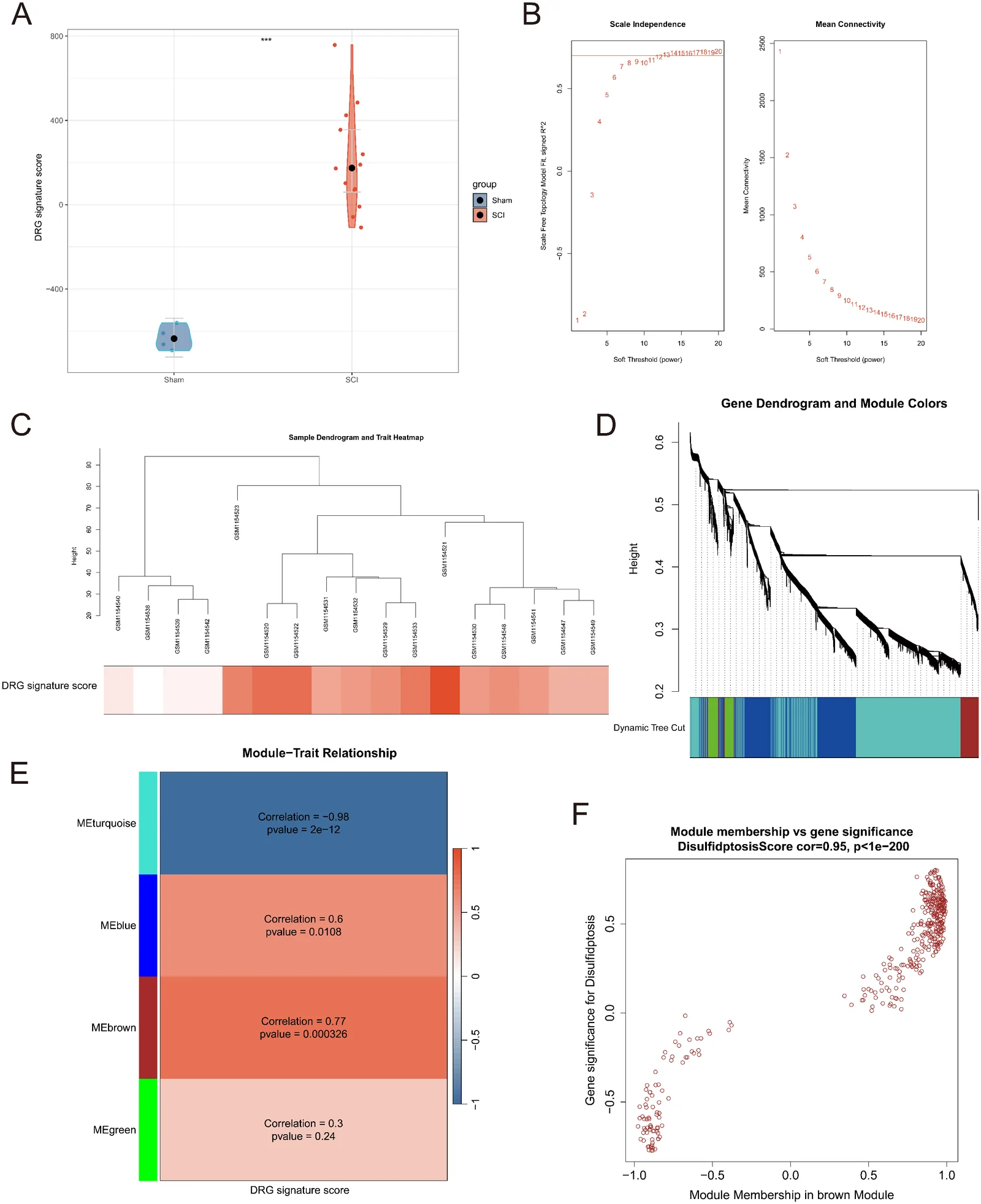
Identification of DRG signature-associated co-expression modules through WGCNA. **(A)** Comparison of DRG signature scores between SCI and Sham groups. **(B)** Selection of the soft-thresholding power based on scale-free topology fit index (R²) and mean connectivity. A soft-thresholding power of 13 was selected for network construction. **(C)** Sample clustering dendrogram and trait heatmap based on bulk transcriptome data. **(D)** Gene dendrogram and module assignment obtained through hierarchical clustering, with different modules indicated by distinct color. **(E)** Heatmap showing correlations between module eigengenes and DRG signature score. **(F)** Scatter plot showing the correlation between MM and GS in the brown module for the DRG signature score.

Using the expression profiles of the predefined 39 DRGs, a PCA-based DRG signature score was calculated to quantify sample-level variation associated with the DRG set across GSE47681 samples. The DRG signature score was significantly elevated in the SCI group compared the Sham controls (**Figure 5A**). WGCNA was subsequently performed using bulk transcriptomic profiles, with the DRG signature score treated as a continuous trait to identify gene co-expression modules associated with the DRG signature. The top 25% most variable genes were selected for primary network construction. A soft-thresholding power of 13 was selected based on a balance between scale-free topology fitting (R² = 0.70) and preservation of sufficient network connectivity (**Figure 5B**; **Supplementary Figure 3**). Sample clustering revealed no obvious outliers, and the distribution of DRG signature scores was consistent across samples (**Figure 5C**). Multiple gene co-expression modules were identified using hierarchical clustering and dynamic tree cutting (**Figure 5D**). Among these modules, the brown module exhibited the strongest correlation with the DRG signature score (Cor = 0.77, *P* = 0.000326) (**Figure 5E**) and was selected as the DRG signature-associated co-expression module for subsequent analyses. Within the brown module, GS and MM were strongly correlated (Cor = 0.95, *P* < 0.001), indicating that highly connected genes within this module were closely associated with the DRG signature score (**Figure 5F**).The 398 genes contained in the brown module were subsequently subjected to GO and KEGG enrichment analyses, revealing significant enrichment in lysosome-related pathways, efferocytosis, extracellular matrix organization, collagen-containing extracellular matrix structures, and cholesterol biosynthetic processes (**Supplementary Figure 4**).

To further evaluate the robustness of the WGCNA network construction and the influence of gene filtering thresholds, sensitivity analyses were performed by reconstructing independent WGCNA networks using the top 15%, 25%, and 50% most variable genes. Although module color labels differed among independently constructed networks, the phenotype-associated modules consistently showed strong associations with the DRG signature score (absolute module–trait correlation coefficients ranging from 0.98 to 0.99, all *P* < 0.001). Furthermore, substantial gene-level overlap was observed among corresponding phenotype-associated modules identified under different filtering strategies, with Jaccard indices ranging from 0.55 to 0.70 (**Supplementary Table 3**). These findings support the robustness of the identified DRG signature-associated co-expression module across different gene filtering strategies.

### 3.5 Analysis and functional profiling of DEGs associated with disulfidptosis

**Figure 6:**
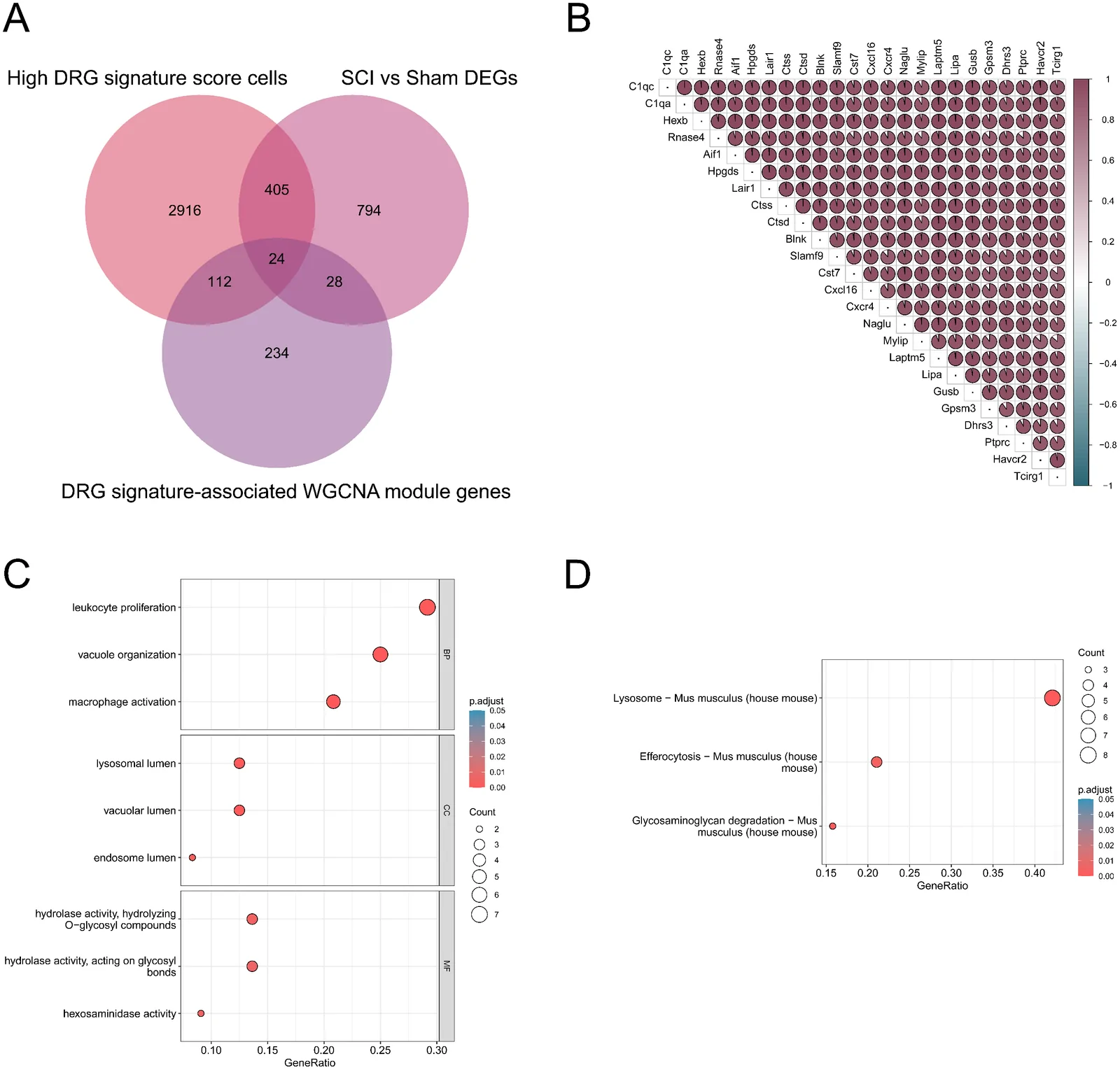
Functional characterization of intersected genes identified from integrative analysis. **(A)** Venn diagram showing overlapping genes among SCI-related DEGs, genes differentially expressed between high- and low-DRG signature score cells, and genes within the WGCNA brown module. **(B)** Spearman correlation analysis of pairwise relationships among the intersected genes. **(C)** GO functional enrichment analysis of the intersected gene set, highlighting enriched biological processes, cellular components, and molecular functions. **(D)** KEGG pathway analysis of the intersected gene set showing associated signaling and metabolic pathways.

To identify genes consistently associated with SCI-related transcriptional alterations and the disulfidptosis-related gene signature, we performed an integrative overlap analysis of DEGs between SCI and Sham samples, genes differentially expressed between high- and low-DRG signature cells, and genes within the WGCNA DRG signature–associated module. This analysis identified 24 intersecting genes (**Figure 6A**). Correlation analysis showed that these genes exhibited coordinated expression patterns (**Figure 6B**). Functional enrichment analysis was subsequently performed to explore their potential biological roles. GO analysis indicated that these genes were primarily enriched in immune-related processes such as macrophage activation and immune cell proliferation. In terms of cellular components, they were associated with lysosomal lumen, endosomal membrane, and vacuolar structures, while molecular function was enriched in hydrolase activity, particularly glycosyl bonds hydrolysis (**Figure 6C**). KEGG pathway analysis further indicated enrichment in lysosomal-related pathways, glycosaminoglycan degradation, and ferroptosis-associated pathways (**Figure 6D**). These results suggest that the identified gene set reflects SCI-related immune and lysosomal responses rather than defining a specific disulfidptosis regulatory program.

### 3.6 Prioritization and external validation of candidate genes

**Figure 7:**
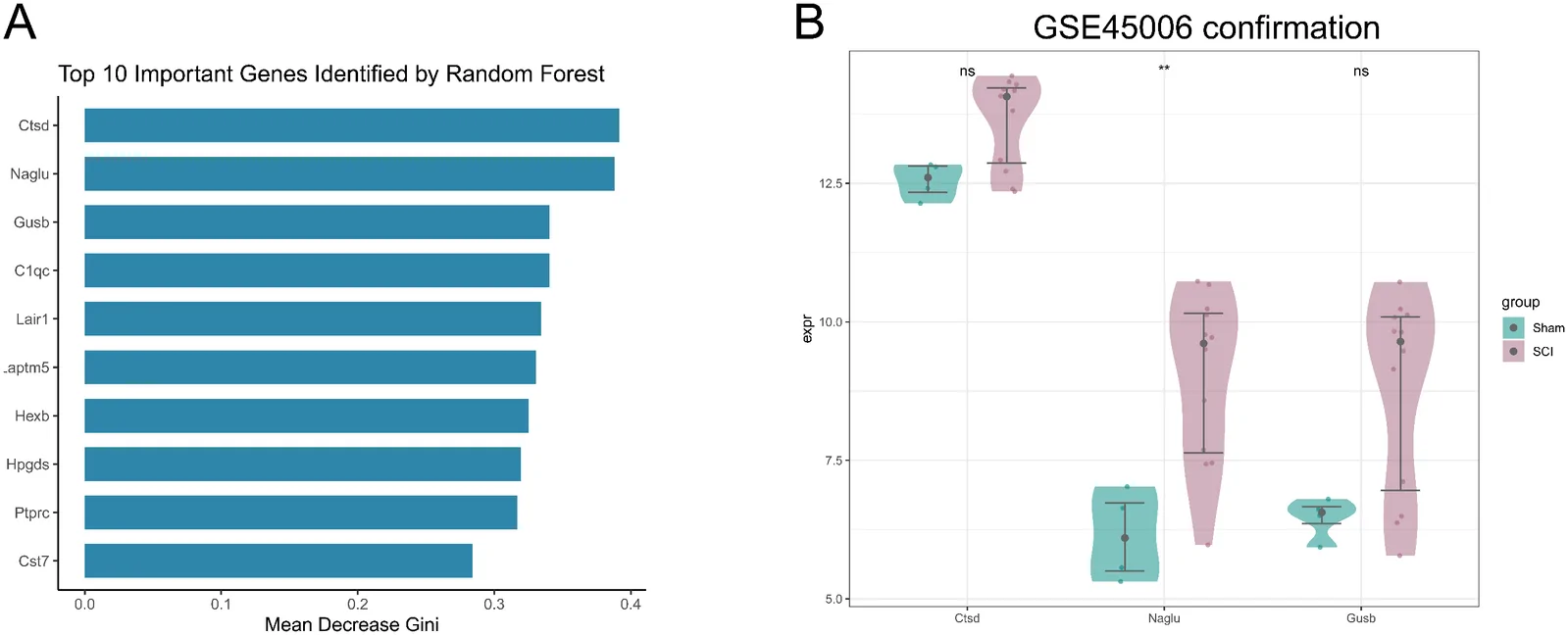
Prioritization and external validation of RF-ranked candidate genes. **(A)** Top 10 integrative analysis-derived candidate genes ranked according to their Mean Decrease Gini values in the RF model, reflecting their relative importance in distinguishing SCI and Sham samples. **(B)** Violin plots showing the expression levels of the three highest-ranked candidate genes (*Ctsd*, *Naglu*, and *Gusb*) in Sham and SCI samples from the independent dataset GSE45006. *Naglu* showed significant differential expression between SCI and Sham samples, whereas *Ctsd* and *Gusb* did not reach statistical significance. Statistical significance is indicated as ns (not significant) or **P < 0.01.

To further prioritize integrative analysis-derived candidate genes, a RF model was applied to a biologically pre-filtered candidate gene set derived from the intersection of genes differentially expressed between SCI and Sham samples, genes differentially expressed between high- and low-DRG signature cells, and genes within the DRG signature-associated co-expression module identified by WGCNA. Rather than serving as a *de novo* gene discovery approach, the RF model was used to rank candidate genes according to their relative contribution to distinguishing SCI and Sham samples. Gene importance was assessed based on the Mean Decrease Gini index. The top 10 ranked genes included *Ctsd*, *Naglu*, *Gusb*, *C1qc*, *Lair1*, *Laptm5*, *Hexb*, *Hpgds*, *Ptprc*, and *Cst7* (**Figure 7A**). To perform external validation of the RF-ranked candidates, the three highest-ranked genes (*Ctsd*, *Naglu*, and *Gusb*) were further examined in an independent transcriptomic dataset (GSE45006). Among these candidates, *Naglu* showed consistent and significant differential expression between SCI and Sham samples, whereas *Ctsd* and *Gusb* did not reach statistical significance (**Figure 7B**). Therefore, *Naglu* was selected for subsequent analyses as the candidate gene supported by the most consistent evidence across computational prioritization and external validation, while *Ctsd* and *Gusb* were considered additional RF-prioritized candidates requiring further investigation.

### 3.7 Single-cell expression pattern of *Naglu* across cell populations

**Figure 8:**
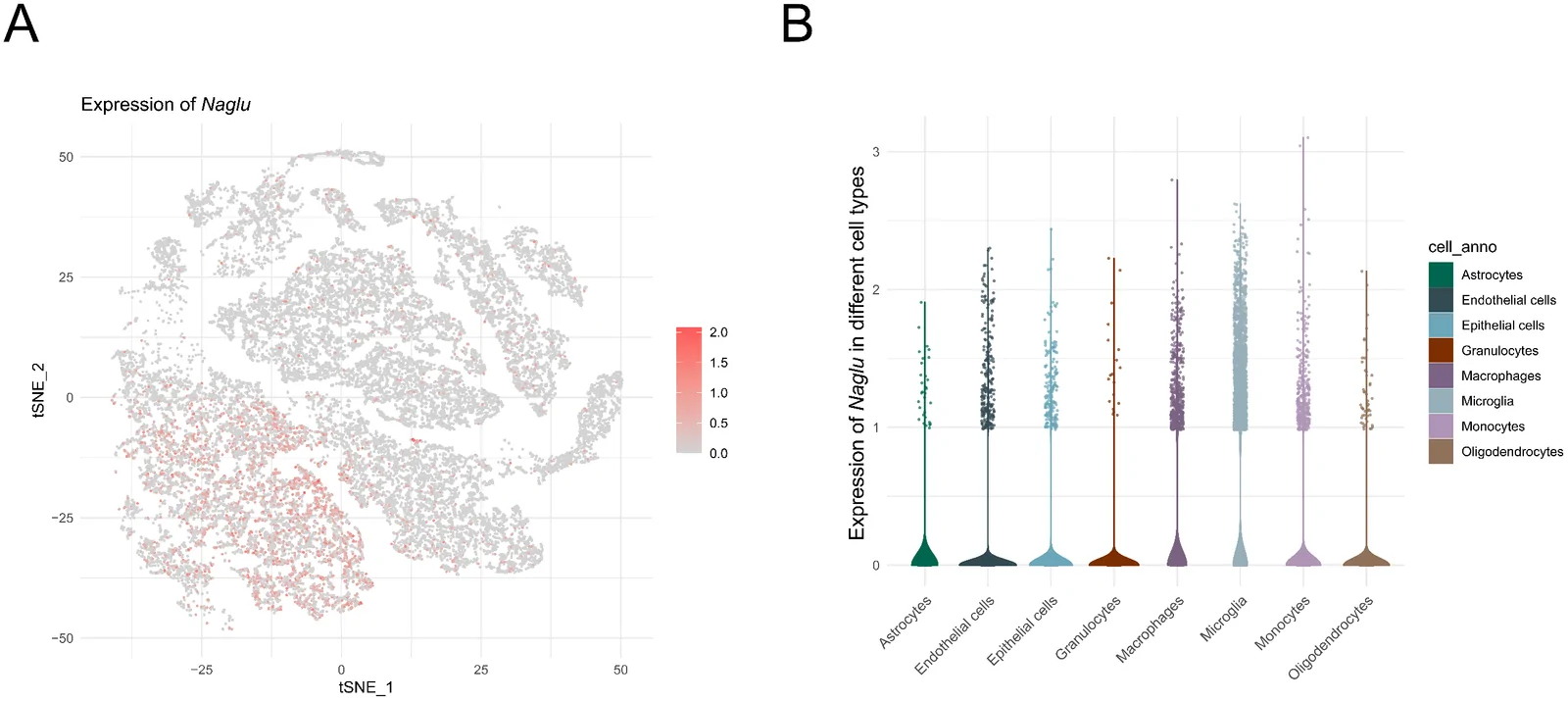
Cell type–specific expression of *Naglu*. **(A)** t-SNE plot of *Naglu* expression in single cells, with color intensity representing expression levels. **(B)** Violin plot depicting the expression profile of *Naglu* across annotated cellular populations.

To characterize the cellular expression pattern of *Naglu*, its expression was examined at the single-cell level. *Naglu* expression was heterogeneous across cells, with higher levels observed in specific clusters (**Figure 8A**). Cell-type annotation revealed that *Naglu* was broadly expressed across multiple populations, with relatively higher expression in microglia and macrophages and relatively lower expression observed in astrocytes and oligodendrocytes (**Figure 8B**). For comparison, the single-cell expression patterns of other RF-prioritized genes (*Ctsd* and *Gusb*) across cell types are shown in **Supplementary Figure 4**. These findings indicate that *Naglu* exhibits a heterogeneous, cell type-associated expression pattern within the SCI microenvironment.

### 3.8 Validation of NAGLU protein expression in rats at 3 days post-injury

**Figure 9:**
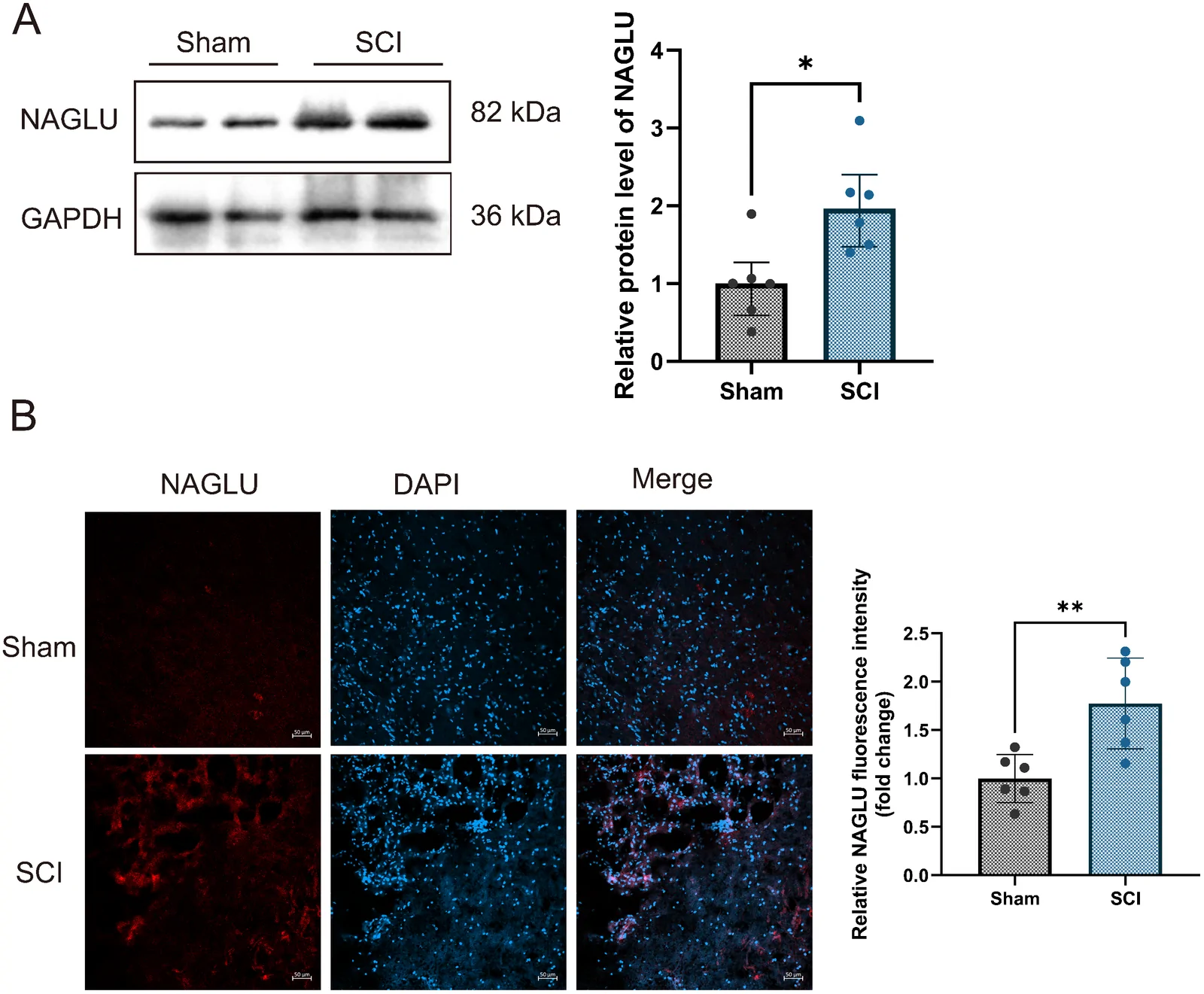
Validation of NAGLU protein expression in a rat SCI model at 3 days post-injury. **(A)** Western blot analysis was performed to evaluate NAGLU protein expression in spinal cord tissues collected from Sham and SCI rats at 3 days post-injury, with GAPDH used as the loading control. Band intensities were quantified and normalized to GAPDH. Data are presented as mean ± SD (n = 6), *P < 0.05 versus the Sham group. **(B)** Immunofluorescence staining was performed to detect NAGLU (red) in spinal cord tissues collected from Sham and SCI rats at 3 days post-injury, with nuclei counterstained with DAPI (blue). Representative images and merged views are shown. NAGLU fluorescence intensity was quantified using ImageJ software. Data are presented as mean ± SD (n = 6). **P < 0.01 versus the Sham group. Scale bar = 50 μm.

Protein-level validation of NAGLU expression was performed in spinal cord tissues collected at 3 days post-injury from Sham and SCI rats. Western blot analysis demonstrated increased NAGLU protein expression in SCI tissues compared with the Sham group (**Figure 9A**). Consistently, immunofluorescence staining showed enhanced NAGLU immunoreactivity in SCI tissues, whereas relatively lower NAGLU immunoreactivity was observed in Sham controls (**Figure 9B**). These findings support the computational identification of NAGLU as an SCI-associated candidate gene and confirm its increased protein expression following SCI. However, these results demonstrate changes in NAGLU expression rather than direct activation of disulfidptosis or a functional regulatory role of NAGLU in this cell death process.

## 4 Discussion

We systematically investigated DRG signature associated with SCI using integrated single-cell transcriptomics, bulk RNA sequencing, random forest modeling, and experimental validation. Our findings provide a multi-level characterization of DRG-associated transcriptional alterations in SCI and reveal substantial heterogeneity in DRG signature enrichment across different cellular populations within the injured microenvironment.

Secondary injury following SCI involves a complex cascade of pathological events, including neuroinflammation, oxidative stress, vascular impairment, and metabolic disturbances (21). Emerging forms of regulated cell death, including ferroptosis and other stress-related pathways, have increasingly been implicated in SCI progression (22, 23). Disulfidptosis is a recently identified form of regulated cell death characterized by excessive disulfide accumulation and disruption of the actin cytoskeleton, resulting in metabolic and redox disturbances (24). However, the transcriptional characteristics of DRGs and their potential association with SCI-associated cellular stress responses remain largely unexplored.

At the single-cell level, DRG signature scores exhibited distinct distribution patterns across cell types. Higher DRG signature scores were observed in granulocytes and endothelial cells, suggesting that DRG-associated transcriptional signatures vary among cellular populations following SCI (25–28). These findings highlight the heterogeneity of DRG signature enrichment across different cell types within the injured spinal cord microenvironment. Because the AUCell-derived score reflects the relative enrichment of a predefined DRG set rather than direct biological activity, these results should be interpreted as cell type-specific transcriptional associations rather than direct evidence of disulfidptosis occurring in these cell populations.

By integrating bulk SCI-associated DEGs, single-cell DEGs identified between high- and low-DRG signature score cells, and genes from DRG signature-associated WGCNA modules, candidate genes associated with DRG-related transcriptional alterations after SCI. Functional enrichment analysis revealed associations with lysosome-related pathways, macrophage activation, and glycosaminoglycan metabolism. Lysosomes play central roles in intracellular degradation, autophagic flux, and cellular stress adaptation and have been implicated in injury-related responses (29, 30). Pathways associated with macrophage activation were also significantly enriched, suggesting immune-related transcriptional alterations, as activated macrophages and microglia contribute to both inflammatory and reparative responses after SCI (31). Collectively, these findings suggest that DRG signatures are associated with transcriptional programs involving immune regulation and lysosomal processes within the SCI microenvironment, providing potential insights into cellular responses following SCI.

Among the enriched biological processes, pathways related to macrophage activation, efferocytosis, and lysosomal function were particularly noteworthy. Efferocytosis is an essential process through which macrophages and microglia clear apoptotic cells and cellular debris after central nervous system injury, thereby facilitating inflammation resolution and tissue repair (32, 33). Following engulfment, apoptotic cells are degraded within phagolysosomes through lysosome-dependent enzymatic processes, making functional lysosomal activity essential for efficient efferocytosis (34).

To further prioritize candidate genes identified through the integrative analysis, a RF model was applied to rank candidate genes according to their relative contribution to distinguishing SCI and Sham samples. Although the RF model showed stable internal cross-validation performance, the relatively small sample size and high feature-to-sample ratio may increase the risk of overfitting. Therefore, the RF analysis was interpreted primarily as a candidate gene prioritization approach rather than as a standalone predictive model or evidence of causal gene involvement. Among the three top-ranked candidate genes, *Naglu*, which encodes α-N-acetylglucosaminidase, showed consistent validation in an independent dataset (GSE45006), supporting *Naglu* as the candidate with the most consistent evidence across computational prioritization and external validation.

In contrast, *Ctsd* and *Gusb*, although highly ranked by the RF model, were not validated in the independent GSE45006 dataset. Several factors may contribute to this discrepancy. First, the discovery and validation datasets were generated using different transcriptomic platforms and different species (mouse versus rat), which may introduce technical and biological variability. Second, differences in injury models, sampling time points, and biological heterogeneity may influence the reproducibility of gene expression patterns across independent cohorts. Finally, because the RF analysis was performed within a relatively small candidate gene set, some highly ranked genes may reflect dataset-specific transcriptional characteristics rather than universally conserved SCI-associated signatures. Therefore, further validation in larger independent cohorts and functional experiments will be required to clarify the biological relevance of *Ctsd* and *Gusb*.

NAGLU is a lysosomal hydrolase involved in heparan sulfate degradation (35). Accordingly, altered *Naglu* expression may reflect changes in lysosomal homeostasis and injury-associated cellular responses after SCI (36, 37). Single-cell transcriptomic analysis revealed a cell-type-specific distribution of *Naglu*, with relatively prominent expression in microglial and macrophage populations. This pattern is biologically plausible because microglia and infiltrating macrophages are major phagocytic populations within the injured spinal cord and contribute to debris clearance, inflammatory regulation, lesion organization, and tissue remodeling after SCI (38, 39). More broadly, the microglial endolysosomal system links intracellular degradation with inflammatory regulation, and lysosomal dysfunction may impair phagocytic and autophagic processing while contributing to persistent neuroinflammatory responses (40, 41).

Notably, granulocytes and endothelial cells exhibited higher overall DRG signature scores, whereas *Naglu* expression was more prominent in microglia. This lack of concordance indicates that *Naglu* expression is not simply proportional to the magnitude of the overall DRG signature across cell types. Instead, *Naglu* may represent a more cell-type-selective lysosomal transcriptional feature associated with microglial and macrophage responses in the injured spinal cord.

Experimental validation demonstrated increased NAGLU protein expression in SCI tissue by Western blotting and immunofluorescence, consistent with the transcriptomic upregulation of *Naglu*. However, Zhang et al. (42) reported decreased NAGLU enzymatic activity after SCI. These observations are not necessarily contradictory because protein abundance and enzymatic activity represent distinct biological parameters. Increased NAGLU protein expression does not necessarily indicate enhanced lysosomal enzymatic function. Under conditions of lysosomal stress, increased lysosomal enzyme abundance may not translate into improved catalytic activity because impaired intracellular trafficking and altered lysosomal homeostasis may affect enzyme localization and function (43, 44).

Although *Naglu* was identified through an integrative prioritization strategy based on DRG-associated transcriptional patterns, the present study does not provide direct evidence that *Naglu* functions as a component or regulator of disulfidptosis. Therefore, *Naglu* should be interpreted as a DRG signature-associated lysosomal gene reflecting injury-related microglial and immune-associated transcriptional states, rather than a confirmed mediator of disulfidptosis. Further studies incorporating cell-type-specific localization, NAGLU enzymatic activity assays, lysosomal functional measurements, and substrate quantification will be required to determine whether NAGLU actively contributes to neuroinflammatory responses after SCI or primarily represents a marker of altered lysosomal homeostasis.

Despite these findings, several limitations should be acknowledged. First, candidate gene identification was primarily based on integrative computational analyses, whereas experimental validation was limited to confirming increased NAGLU protein expression. Consequently, the causal roles of NAGLU and other identified candidate genes and pathways in SCI progression remain uncertain and require validation through functional perturbation experiments. Second, although DRG signature-associated transcriptional patterns were characterized, their functional significance and underlying mechanisms remain to be elucidated. In particular, whether NAGLU actively regulates microglial activation and lysosomal function or merely reflects injury-associated lysosomal responses remains unclear. Gain- and loss-of-function studies, together with direct assessment of lysosomal activity, microglial phenotypes, and cell-death-related changes, will be required to clarify these relationships. Third, samples collected at 1, 3, and 7 days post-injury were pooled for the bulk transcriptomic analyses rather than analyzed separately by time point. Therefore, the present study cannot distinguish time-dependent molecular changes or determine whether *Naglu* expression follows a transient or sustained pattern after SCI. Moreover, *in vivo* validation experiments were conducted exclusively at 3 days post-injury. Future studies incorporating multiple post-injury time points are needed to characterize the temporal expression pattern and functional relevance of NAGLU during SCI progression. Fourth, the relatively small number of animals used for experimental validation and the exclusive use of female rats may limit the generalizability of the findings. Although female rats were used to reduce biological variability within the experimental cohort, sex-dependent effects related to hormonal regulation, inflammatory responses, and tissue repair after SCI cannot be excluded. Future studies including both male and female animals are needed to determine whether the observed molecular changes are sex-specific or broadly applicable. Fifth, DRG signature-associated transcriptional states were inferred using a predefined gene signature rather than assessed through direct experimental measurements of disulfidptotic cell death. Accordingly, the AUCell-derived DRG signature score should be interpreted as reflecting the relative enrichment of a predefined DRG set rather than direct evidence for the occurrence or biological activity of disulfidptosis. Furthermore, because AUCell does not account for the directionality or functional contribution of individual genes within the predefined gene set, these scores represent transcriptional associations rather than direct functional measurements of disulfidptosis. In addition, the observed correlation between DRG and HSP-related signatures suggests that some DRGs may capture shared cellular stress programs, further emphasizing the need for cautious interpretation of the DRG signature score and future experimental validation of disulfidptosis execution. Finally, although immunofluorescence analysis confirmed increased NAGLU immunoreactivity in injured spinal cord tissue, the present study did not determine its precise cell-type-specific localization or functional contribution to SCI-associated cellular responses. Cell-type-specific colocalization analyses, conditional genetic manipulation, and mechanistic experiments are therefore required to further define the biological role of *Naglu* in SCI.

## 5 Conclusions

This study provides a comprehensive characterization of DRG signature-associated transcriptional alterations in SCI and identifies *Naglu* as a microglia-enriched candidate gene linked to DRG signatures. Functional enrichment analyses highlighted lysosome-related pathways, efferocytosis, and immune-associated processes, suggesting a potential association between DRG signature enrichment and lysosomal responses following SCI. These findings provide a foundation for future mechanistic studies investigating how DRG signature-associated cellular responses may contribute to neuroimmune remodeling and repair-associated processes after SCI.

## Data Availability

The datasets presented in this study can be found in online repositories. The names of the repository/repositories and accession number(s) can be found in the article/**Supplementary Material**.
