# Supplementary material for: Integrative single-cell and bulk transcriptomic analyses identify a microglia-associated NAGLU signature linked to disulfidptosis-associated transcriptional patterns after spinal cord injury
Source: Front Immunol. 2026 Aug 14;17:1899070. doi: 10.3389/fimmu.2026.1899070 (PMC13522192; doi:10.3389/fimmu.2026.1899070)
Supplement: Supplementary file 1 [file DataSheet1.docx]

Supplementary Figure S1. Temporal dynamics of cellular composition and DRGs expression. (A) Changes in the absolute numbers of annotated cell populations across different post-injury time points (Uninjured, 1 dpi, 3 dpi, and 7 dpi). (B) Relative proportions of each cell population at different time points. (C) t-SNE visualization of annotated cell populations at each post-injury time point, showing the temporal distribution of major cell types. (D) Dot plot showing the expression patterns of DRGs across different post-injury time points. Dot size represents the percentage of cells expressing each gene, and color intensity indicates the average expression level.

Supplementary Figure S2. Evaluation of stress-related features associated with the DRG signature using a heat shock-related gene signature. (A) Distribution of HSP-related signature scores across annotated cell types. (B) t-SNE visualization of HSP-related signature scores across single cells. (C) Correlation analysis between DRG signature scores and HSP-related signature scores.

Supplementary Figure S3. Evaluation of soft-thresholding power selection for WGCNA under different highly variable gene selection thresholds. Scale-free topology fit index (signed R²) and mean connectivity were evaluated across a range of soft-thresholding powers (β = 1–20) using different highly variable gene filtering strategies. (A) WGCNA parameter evaluation based on the top 15% most variable genes. (B) WGCNA parameter evaluation based on the top 25% most variable genes used for the primary network construction. (C) WGCNA parameter evaluation based on the top 50% most variable genes. The vertical dashed line indicates the soft-thresholding power used for downstream network construction (β = 13), which was selected based on a balance between scale-free topology fitting and preservation of sufficient network connectivity.

Supplementary Figure S4. Functional enrichment analysis of the brown module identified from the primary WGCNA analysis. (A) GO enrichment analysis of genes within the brown module, including biological process (BP), cellular component (CC), and molecular function (MF) categories. (B) KEGG pathway enrichment analysis of genes within the brown module.

Supplementary Figure S5. Expression patterns of *Ctsd* and *Gusb* in the single-cell RNA-seq dataset. (A, C) t-SNE plots showing the expression distribution of *Cts*d and *Gusb* across all cells in the spinal cord injury scRNA-seq dataset. Each dot represents a single cell, and color intensity indicates normalized gene expression levels. (B, D) Violin plots showing the expression levels of *Ctsd* and *Gusb* across annotated cell types in the dataset.
